# The Railmap of Type I Interferon Induction: Subcellular Network Plan and How Viruses Can Change Tracks

**DOI:** 10.3390/cells11193149

**Published:** 2022-10-06

**Authors:** Laura Weber, Gabrielle Vieyres

**Affiliations:** 1 Junior Research Group “Cell Biology of RNA Viruses”, Leibniz Institute of Virology, 20251 Hamburg, Germany; 2 Integrative Analysis of Pathogen-Induced Compartments, Leibniz ScienceCampus InterACt, 20251 Hamburg, Germany

**Keywords:** innate immunity, type I interferon, spatiotemporal organization, subcellular compartmentalization, organelle, viral antagonism, viral evasion, pattern-recognition receptor, adaptor, supramolecular organizing center (SMOC)

## Abstract

The innate immune response constitutes the cell’s first line of defense against viruses and culminates in the expression of type I interferon (IFN) and IFN-stimulated genes, inducing an antiviral state in infected and neighboring cells. Efficient signal transduction is a key factor for strong but controlled type I IFN expression and depends on the compartmentalization of different steps of the signaling cascade and dynamic events between the involved compartments or organelles. This compartmentalization of the innate immune players not only relies on their association with membranous organelles but also includes the formation of supramolecular organizing centers (SMOCs) and effector concentration by liquid–liquid phase separation. For their successful replication, viruses need to evade innate defenses and evolve a multitude of strategies to impair type I IFN induction, one of which is the disruption of spatial immune signaling dynamics. This review focuses on the role of compartmentalization in ensuring an adequate innate immune response to viral pathogens, drawing attention to crucial translocation events occurring downstream of pattern recognition and leading to the expression of type I IFN. Furthermore, it intends to highlight concise examples of viral countermeasures interfering with this spatial organization to alleviate the innate immune response.

## 1. Introduction

Compartmentalization subtends most biological processes at the cellular level. While subcellular organization in distinct compartments is particularly refined in eukaryotes, even prokaryotes are now accepted to segregate functions in protein- or membrane-bound organelles [1]. A compartmentalized cell plan allows the co-existence of distinct microenvironments with tightly regulated properties, such as pH or redox status, enabling specialized functions. It has also evolved to physically separate and coordinate in time and space multiple biochemical activities, even conflicting ones such as protein synthesis and degradation, to manage reserves and to confine toxic molecules [2]. Coordination of segregated activities requires communication, which is ensured by vesicular and non-vesicular (diffusion-mediated) trafficking between compartments. In the last decade, special attention has been paid to membrane contact sites, which mediate communication and exchanges between closely apposed organelles [3]. In parallel, a growing number of non-canonical organelles have been identified that are not limited by a phospholipid bilayer but consist of dynamic and membrane-less macromolecular condensates assembled by liquid–liquid phase separation [4]. These new findings have revolutionized and, at the same time, blurred our textbook picture of the eukaryotic cell plan.

The type I interferon (IFN) system belongs to the first line of defense protecting all nucleated cells against viral infections and can be divided into two signaling cascades, IFN induction and IFN response [5]. IFN induction relies on the specific detection of conserved pathogen-associated molecular patterns (PAMPs) by a range of pattern-recognition receptors (PRRs) and on a signaling cascade from the cell periphery (plasma membrane or endosomal network) to the nucleus. Signals from different PRRs converge using a handful of adaptor proteins and are further funneled into the usage of a common set of serine/threonine kinases and transcription factors such as the IFN regulatory factors (IRFs) IRF3 and IRF7. Nuclear translocation of these transcription factors induces a panel of host defense effectors, including IFNs and a range of pro-inflammatory cytokines [5]. Type I IFNs are secreted proteins that bind the heterodimeric IFN α and β receptor subunit 1/2 (IFNAR1/IFNAR2) receptor complex in an autocrine and paracrine manner. The subsequent signal follows the Janus kinase (JAK)/ signal transducer and activator of transcription (STAT) pathway to induce the nuclear translocation of the IFN-stimulated gene factor 3 (ISGF3) transcription complex and regulate a number of genes and non-coding RNAs. Target genes include, in particular, a myriad of IFN-stimulated genes (ISGs), many of them having antiviral functions [6]. With PRRs at the cell periphery, adaptor molecules anchored on intracellular membranes, and a centripetal signaling cascade resulting in nuclear translocation of transcription factors, the IFN gene induction pathway is tightly regulated in time and space and heavily relies on host cell compartmentalization and on protein dynamics between compartments [7].

Viral infection often results in a turmoil in the cell, hijacking resources and reprogramming the host for their own benefit. Strikingly, many viruses organize their replication organelle by remodeling host cell membranes or forming membrane-less condensates, again highlighting the functional importance of subcellular compartmentalization [8]. In an evolutionary arms race with the host, viruses also developed multiple ways to blunt the cell’s innate immune response and antagonize IFN production [9,10].

This review concentrates on the subcellular compartmentalization of type I IFN induction in response to viral infection. It also describes how viruses can block IFN induction by disrupting this spatial organization by sequestrating, retargeting, or even repurposing innate immune factors. For clarity, it focuses on the main innate immune sensors, adaptors, and effectors and highlights selected examples of cellular compartmentalization and viral antagonism. Therefore, it does not have the presumption to provide an exhaustive picture of the molecular pathways leading to IFN production or of the many viral evasion strategies. Rather, it intends to emphasize common themes and concepts in innate immune compartmentalization while encompassing the newly acknowledged complexity in subcellular architecture and dynamics.

## 2. Spatial Distribution of Antiviral Innate Immune Signaling Cascades

### 2.1. Virus Sensing Is Tightly Compartmentalized

A key feature for rapid recognition of PAMPs is the strategic localization of PRRs within the cell and on the cell surface. This allows immediate access to the ligand at the site where it first becomes accessible. While highly conserved structures displayed on the surface of bacteria and fungi can be detected by Toll-like receptors (TLRs) on the cell surface [11], recognition of viruses mainly relies on their nucleic acid genomes as the most potent PAMPs, and therefore, elicitation of an innate immune response mostly originates from intracellular receptors. Most PRRs for viral nucleic acids reside in the cytosol or within endosomes. Recent evidence, however, suggests that this textbook picture might be too simplified and rather highlight further layers of compartmentalization, with the sensors often able to shuttle between multiple locations within the cell and/or to concentrate in subdomains rather than homogenously spreading in the whole compartment.

#### 2.1.1. Detection of Viral Nucleic Acids in the Cytoplasm

**Figure 1:**
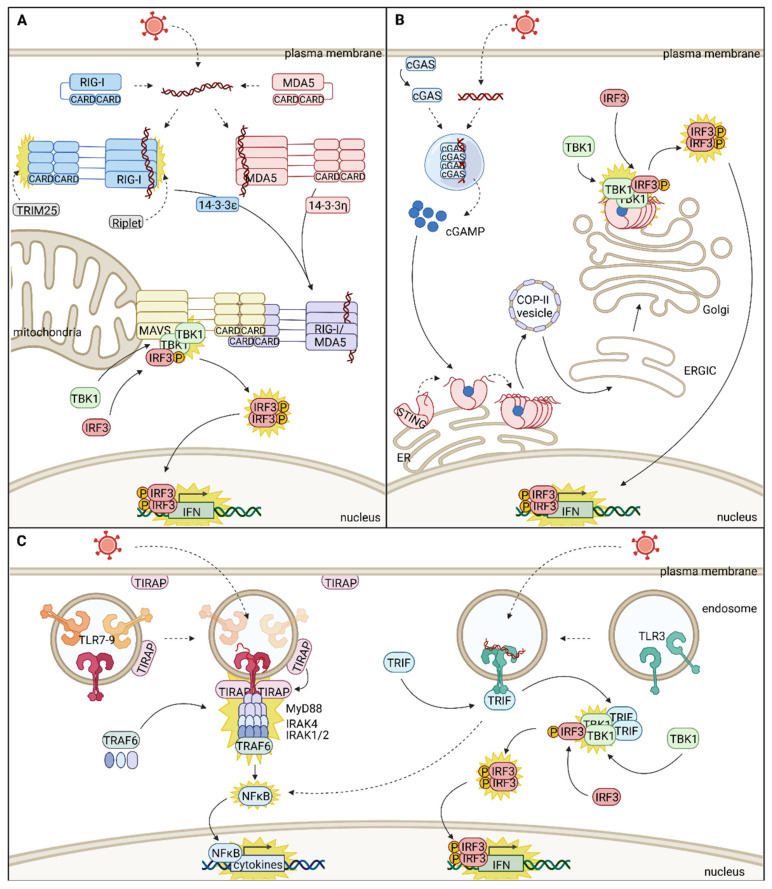
Compartmentalization and spatial dynamics of innate immune signaling pathways inducing nuclear translocation of IRF3 and IFN expression. (**A**) Viral double-stranded RNA in the cytoplasm is sensed by retinoic acid-inducible gene-I (RIG-I) and melanoma differentiation gene 5 (MDA5), causing caspase recruitment domain (CARD)-mediated oligomerization of the receptors. RIG-I oligomers are further activated through posttranslational modifications introduced by the E3 ubiquitin ligases tripartite motif containing 25 (TRIM25) and Riplet. Translocation of activated receptor oligomers to sites of mitochondrial antiviral signaling (MAVS) localization (mitochondria, mitochondria-associated membranes, and peroxisomes) is guarded by 14-3-3 chaperones. CARD-mediated interactions activate MAVS and cause the recruitment and autophosphorylation of TANK-binding kinase 1 (TBK1) and, subsequently, phosphorylation of interferon regulatory factor 3 (IRF3). Phosphorylated IRF3 forms dimers and translocates into the nucleus, where it functions as transcription factor to induce the expression of interferon (IFN) and a subset of IFN-stimulated genes (ISGs). (**B**) In the inactive state, cyclic GMP-AMP synthase (cGAS) resides at the inner leaflet of the plasma membrane and shuttles to the cytoplasm upon activation. cGAS senses cytoplasmic viral DNA and utilizes it as template to induce liquid–liquid phase separation to facilitate the production of the second messenger cGAMP. cGAMP binds to inactive stimulator of IFN genes (STING) dimers residing at the ER and induces conformational changes that enable oligomerization of STING. STING then continues to traffic through several membranous compartments, from the ER via COP-II coated vesicles to the ERGIC and Golgi, where it is fully activated by further posttranslational modification. STING recruits TBK1, which is activated by autophosphorylation and continues to phosphorylate IRF3. Phosphorylated IRF3 forms dimers and translocates into the nucleus to induce the expression of IFN and a subset of ISGs. (**C**) Viral single-stranded RNA and CpG containing DNA within endosomes can be sensed by Toll-like receptor (TLR)7/8 and TLR9, respectively. Sensing of a viral pathogen-associated molecular pattern (PAMP) causes conformational changes resulting in dimerization of the signaling domains. The adaptor protein TIRAP resides at both the inner leaflet of the plasma membrane and the cytoplasmic face of the endosome, where it screens for activated TLRs. Binding of TIRAP to TLR signaling domains results in the recruitment/assembly of the myddosome, consisting of MyD88, interleukin-1 receptor-associated kinase 4 (IRAK4), and IRAK1/2 oligomers, and recruitment of TRAF6 to mediate nuclear factor-κB (NFκB) activation. Activated NFκB translocates into the nucleus and functions as transcription factor to induce the expression of cytokines. Besides its ability to activate NFκB, TLR3 is also capable of inducing IFN expression by activation of IRF3. In the inactive state, TLR3 resides as monomer within endosomes and dimerizes only upon activation. Activation of TLR3 by endosomal viral dsRNA results in the recruitment of TRIF. However, TRIF and TLR3 co-localize only transiently, and TRIF continues to form speckle-like cytoplasmic signalosomes with TBK1. Within these signalosomes, TBK1 is activated by autophosphorylation and then phosphorylates IRF3 to enable dimerization, translocation, and induction of IFN and ISG expression. In this figure, re-localization events are indicated with continuous arrows, and activation events are indicated by yellow stars.

The cytoplasmic RNA receptors retinoic acid-inducible gene-I (RIG-I) and melanoma differentiation gene 5 (MDA5), as well as the signaling incompetent laboratory of genetics and physiology 2 (LGP2), constitute the RIG-I-like receptor (RLR) protein family [12]. To prevent recognition of endogenous RNA in the cytoplasm, RIG-I and MDA5 sense specific features of viral genomes or replication intermediates such as 5′ triphosphate RNA or long double-stranded (ds) RNA [7]. Besides their DExH/D box helicase domain and C-terminal domain (CTD) required for RNA binding, RIG-I and MDA5 comprise tandem caspase recruitment domains (CARDs) for signal transduction. RIG-I and MDA5 are kept in an auto-inhibitory state unless viral RNA is bound, which induces massive conformational changes, exposes the CARDs, and removes inhibitory phosphorylation patterns [12] (Figure 1A). Besides binding of viral RNA, changes in the actin cytoskeleton integrity are also key determinants for RLR activation. Substrate specificity of the protein phosphatase-1 (PP1), responsible for CARD dephosphorylation, is conferred by the PP1-regulatory protein PPP1R12C, which resides at the actin cytoskeleton in the absence of infection. Modification of the actin cytoskeleton structure, often induced by viral infection, causes the release of PPP1R12C and enables RLR activation by the mediation of CARD dephosphorylation [13]. Importantly, both MDA5 and RIG-I were shown to form filaments on viral RNA, thus enabling CARD oligomerization and facilitating signal transduction [14,15]. However, posttranslational modifications of RIG-I, namely the attachment of K63-linked ubiquitin polymers by tripartite motif containing 25 (TRIM25) and Riplet, also contribute to the elicitation of downstream signaling [9]. Interestingly, viral infection is often accompanied by the formation of membrane-less cytosolic RNA-protein complexes called antiviral stress granules [16]. These structures are mediated by protein kinase R (PKR); they concentrate viral RNAs, antiviral proteins such as PKR itself or RNaseL but also RIG-I and thereby promote RIG-I signaling, suggesting that stress granules might provide a physical platform for efficient viral RNA sensing in the cytosol [17,18]. In the case of yellow fever virus infection, however, RIG-I incorporation into stress granules takes place but does not seem relevant for its signaling [19].

Besides RNA, viral DNA is also a common PAMP detected by PRRs in the cytoplasm. One very prominent receptor for viral dsDNA in the cytoplasm is the cyclic GMP-AMP synthase (cGAS), which synthesizes the second messenger cGAMP, a non-canonical 2′-5′-linked cyclic dinucleotide [20] (Figure 1B). However, cGAS is not merely cytosolic but shuttles between several compartments with important functional consequences on its antiviral activity. In fact, in the inactive state, cGAS binds PI(4,5)P2 lipids and resides at specific micro-domains of the plasma membrane, called lipid rafts, a process that minimizes self-DNA recognition [21]. Upon activation, cGAS translocates to the cytoplasm, and dsDNA binding induces liquid–liquid phase separation. In the resulting liquid-like droplets, viral DNA is sequestered in confined foci where enzyme and reactant are concentrated. Thus, phase separation greatly amplifies the production of cGAMP and allows a “switchlike” response when the viral DNA concentration exceeds a threshold [22]. Both plasma membrane localization of inactive cGAS and liquid–liquid phase separation of active cGAS are facilitated by the protein’s N-terminus [21,22]. The absence of the N-terminus results in cytoplasmic localization of cGAS and higher basal levels of IFN; however, it also has a weaker response to viral infection [21]. This effect might be caused by impaired liquid–liquid phase separation. Interestingly, cGAS cytosolic foci contain key components of antiviral stress granules, and their formation also requires PKR and the stress granule initiating protein G3BP1 [23]. Finally, many cells also contain significant pools of nuclear cGAS, which fulfills a variety of different functions, including functions relating to antiviral innate immune responses [20]. In particular, nuclear cGAS is involved in the sensing of viral nucleic acids, such as human immunodeficiency virus (HIV) and herpes simplex virus 1 (HSV-1) DNA [24,25]. Furthermore, nuclear cGAS restricts both DNA and RNA virus infections by recruiting Prmt5, which catalyzes histone modification and increases chromatin accessibility on the IFN gene promoters and enhancers, thus elevating their transcription [26].

#### 2.1.2. Detection of Viral Nucleic Acids in Endosomes

Depending on the virus entry or elicitation mechanism, viral nucleic acids are also released within endosomes, where they are detected by different TLRs (Figure 1C). TLRs are a group of membrane glycoproteins comprising a leucine-rich repeat-containing domain facing the endosome for ligand recognition, a transmembrane domain, and a cytosolic Toll-interleukin-1 receptor (TIR) homology domain for signal transduction [27]. Viral ligands of the endosomal TLRs are dsRNA (TLR3), single-stranded (ss) RNA (TLR7 and TLR8), and unmethylated CpG-containing DNA (TLR9). Ligand binding mediates dimerization of the TLR ectodomains, leading to the dimerization of the cytosolic TIR domains, a prerequisite for signal transduction. For endosomal TLRs, correct localization is essential to ensure accurate signaling. TLRs are synthesized at the endoplasmatic reticulum (ER) and access their final destination through the secretory pathway, as reviewed previously [27]. In brief, the ER chaperone Unc93B1 is a major contributor to endosomal TLR targeting and targets the TLRs to coat protein complex (COP)-II coated vesicles. After passing the Golgi complex, TLRs are sorted to the endosomal network, directly or after reinternalization from the plasma membrane, by interaction with various adaptor protein complexes (APs).

Restricted localization of TLRs to the endosomes rather than the plasma membrane is likely essential to preferentially sense non-self nucleic acids. Indeed, self nucleic acids in the extracellular space are usually degraded before reaching the endosomal compartment and therefore do not trigger the endosomal TLRs. On the contrary, foreign nucleic acids are typically protected, for instance, by the viral capsid, and only able to be sensed once released after enzymatic digestion of the virion in the endosomal pathway [28]. The endosomal environment also plays a substantial role in ensuring TLR signaling. However, while endosomal acidification is important for TLR3 signaling [29], this does not apply to TLR9 [28]. Furthermore, several TLRs function by recognizing a combination of nucleic acids and free nucleosides. Consistently, a knockdown of endosomal nucleic acid degrading enzymes impaired endosomal TLR8 and TLR9 signaling [12,30], again highlighting the relevance of correct receptor localization.

#### 2.1.3. PRR Localization Regulates the Quality and Strength of IFN Induction

In addition to the general spatial distribution between plasma membrane and endosomes as described above, signaling from TLRs is highly dependent on cell type and further layers of compartmentalization. Plasmacytoid dendritic cells (pDC) constitute a specialized cell population that selectively expresses TLR7 and TLR9 and secretes high amounts of type I IFN in response to immune stimulation [31]. The first evidence for the impact of endosomal sub-compartments on signaling outcomes was provided by experiments with synthetic ligands of TLR9. Those different ligands exhibit a distinct potential to induce type I IFN production, which depends on their longevity within the endosomal compartment. In contrast to ligands that rapidly enter the lysosome, TLR9 ligands retained in the endosome potently activate IRF7, an event obligatory for subsequent type I IFN expression [32]. In 2010, Sasai et al. were able to specify the underlying mechanism by identification of two TLR9 signaling competent endosome sub-populations. Those sub-populations were termed nuclear factor-κB (NFκB) and IRF7 endosomes in relation to the transcription factors they activate. Post synthesis at the ER, TLR9 traffics to early VAMP3^+^ LAMP2^−^ endosomes. There it is cleaved for activation and mediates NFκB signaling resulting in the expression of pro-inflammatory cytokines. For type I IFN production, TLR9 needs to journey on to LAMP2^+^ lysosome-related organelles (LROs), which exhibit some lysosomal characteristics but are overall distinct from lysosomes. Targeting to LROs is AP-3 dependent and enables IRF7 activation and subsequent type I IFN production. The dependency on TLR9 relocation to LROs is conferred by the restricted localization of TNF receptor-associated factor (TRAF) 3. This signaling constraint can be bypassed by targeting TRAF3 from LROs to early VAMP3^+^ LAMP2^−^ endosomes, thus enabling IRF7 activation from NFκB endosomes [33]. In vivo experiments underlined the relevance of AP-3 for type I IFN production in response to CpG DNA but could not reproduce similar results when using murine cytomegalovirus infection as a trigger for innate immune activation [34]. Likewise, the relevance of specific endosomal compartments for signaling outcome in pDCs was also suggested for TLR7 [32,33] but has not been investigated in detail.

Other immune cells with unique receptor utilization are inflammatory macrophages. TLR2 is a cell surface receptor for viral and bacterial ligands and induces the expression of tumor necrosis factor (TNF) in response to both. In addition, inflammatory macrophages possess the inimitable ability to produce type I IFN in response to viral TRL2 stimuli in a process dependent on internalization of the receptor and myeloid differentiation primary response 88 (MyD88) mediated signal transduction. Inhibition of endocytosis and, consequently, receptor re-localization results in a loss of type I IFN but not TNF production [35], demonstrating that the localization of TLR2 determines the signaling outcome.

Apart from immune cells, also polarized cells such as intestinal epithelial cells (IECs) activate diverse signaling pathways depending on PRR localization. IECs possess an apical membrane that faces the gut lumen and a basolateral membrane facing the lamina propria [36]. While immune stimulation from the apical membrane should be tolerated, stimulation from the basolateral site indicates trespassing of the endothelial barrier and must induce an immune response. IECs have developed different strategies to ensure differential signaling from the polarized surfaces. In contrast to its endosomal localization in immune cells, TLR9 localizes to both the apical and basolateral surface in IECs. However, the response to receptor activation differs depending on the site of activation. Basolateral stimulation of TLR9 results in NFκB activation and cytokine expression, while apical stimulation induces accumulation of the NFκB inhibitor IκBα to keep NFκB in an inactive state. Furthermore, apical stimulation of TLR9 confers tolerance to a secondary basolateral stimulation [36,37]. In contrast to TLR9 signaling, which only produces cytokines in IECs, TLR3 activation also facilitates IFN production. TLR3 specifically localizes to the basolateral membrane of IECs in a process controlled by AP-1. Absence of AP-1 results in localization at and signaling from both the apical and basolateral membrane in response to a synthetic trigger or mammalian reovirus infection [38].

### 2.2. Adaptor Proteins Organize Platforms for Signal Transduction at Specific Organelles or Organelle Contact Sites

The spectrum of PRRs matches the diversity of pathogens to detect, and their subcellular distribution allows surveying the most relevant pathogen entry sites. However, while initiated in different cell compartments, innate immune signaling converges to a limited number of transcription factors, in particular IRF3 and IRF7, to initiate the type I IFN production and NFκB to induce a panel of inflammatory cytokines. These transcription factors are sequestered in the cytosol until immune stimulation induces a range of posttranslational modifications that enable their nuclear translocation. Once in the nucleus, IRF3 and IRF7 bind to the type I IFN promoters and other promoters of a subset of ISGs and thus induce an antiviral state [39] (Figure 1).

A common feature of all these signaling pathways activated by PAMP sensing is the spatial separation of pattern recognition and signal transduction. This might serve as a safety mechanism to regulate immune activation and highlights the importance of adaptor proteins [40]. Therefore, to trigger innate immunity, PRRs need to translocate or send a signal to a handful of adaptor proteins that orchestrate the downstream response by assembling complex protein platforms on signaling-competent organelles. Kagan, Magupalli, and Wu (2014) proposed the concept of supramolecular organizing centers (SMOCs) for these signaling platforms. SMOCS are site-specific, signaling competent higher-order assemblies in which allosteric interactions required for enzyme activation are enhanced by the increased local concentration of signaling components. These higher-order complexes concentrate receptor, adaptor, and effector molecules, and their assembly generally involves nucleated polymerization of at least one of these factors. In fact, except for cGAS, all PRRs directly provide a platform for complex assembly following ligand binding and PRR polymerization. Typically, SMOCs constitute rigid protein scaffolds that localize to the cytosolic surface of membranes [41] or form liquid-like phase separated, membrane-less, condensates [42]. The following paragraphs describe the location of the best-known innate immune signalosomes, built around the mitochondrial antiviral signaling (MAVS), stimulator of IFN genes (STING), MyD88, and TIR-domain-containing adaptor-inducing IFN-β (TRIF) adaptor proteins.

#### 2.2.1. MAVS Signalosome Is Highly Dependent on Mitochondria and Related Organelle Dynamics

The CARD-containing MAVS protein constitutes the signaling adaptor for the RLRs RIG-I and MDA5 [12] (Figure 1A). Besides its mitochondrial localization conferred by its C-terminal domain [43], MAVS was also reported to reside at mitochondria-associated membranes (MAMs) and peroxisomes [44,45]. Therefore, to trigger signal transduction after sensing viral RNA, RIG-I and MDA5 need to translocate to these sites of MAVS localization. It is well established that the translocation of RIG-I is supported by the chaperone 14-3-3ε [46]. More recently, the chaperone 14-3-3η was shown to fulfill similar functions in the redistribution of MDA5. 14-3-3η enhances MDA5 activation and oligomerization, and the loss of this chaperone prevents MDA5 redistribution to the mitochondria-MAM fraction in response to immune elicitation [47]. MDA5 and RIG-I activate MAVS via CARD interactions [12]. The RIG-I CARDs were directly shown to provide a template for MAVS oligomerization by CARD-CARD interactions [15], and the expression of both N-termini of RIG-I or MDA5 is sufficient to induce the formation of large, prion-like MAVS aggregates, which are highly capable of activating IRF3 in cell culture [48]. To recruit IRF3 and fulfill its function as a scaffold for transcription factor activation, MAVS needs to be phosphorylated by TANK-binding kinase 1 (TBK1) and/or IκB kinase (IKK). Upon activation, MAVS recruits TRAF-associated TBK1, resulting in its oligomerization and trans-autophosphorylation. TBK1 and/or IKK then continue to phosphorylate MAVS at a conserved pLxIS residue. Subsequently, phosphorylated MAVS binds to conserved, positively charged residues of IRF3, recruiting it to the protein complex for its phosphorylation and activation by TBK1 [49]. Activated IRF3 dissociates from the protein complex, dimerizes, and translocates into the nucleus, where it acts as a transcription factor and induces IFN expression [39].

MAVS localization and its relevance for signaling outcome, as is the case with TLR localization, is still a matter of debate. Nevertheless, mitochondria association seems to be of great relevance for MAVS-mediated signal transduction since successful signaling is highly dependent on mitochondrial dynamics [50]. Mitochondria are subject to mitochondrial fusion and fission events, and activation of RLR receptors is a trigger for mitochondrial elongation. Further induction of mitochondrial fusion, for example, by silencing of mitochondrial fission effectors, has an overall positive effect on antiviral innate immune signaling. On the opposite, silencing the expression of proteins supporting mitochondrial fusion attenuates the immune response [51]. MAVS directly associates with two key factors of mitochondrial fusion, mitofusin (MFN) 1 and MFN2 [50,51,52]. While MFN1 supports antiviral signaling, MFN2 overexpression causes downregulation of IFN-β [52], thus deviating from the pattern that mitochondrial fusion effectors positively regulate signal transduction. However, this effect could be caused by MFN2’s ability to target MAVS for proteasomal degradation and might not be related to its role in mitochondria fusion [53]. Nevertheless, the DENV protease NS2B3 has evolved to cleave both MFN1 and MFN2 to attenuate immune activation and cytopathy, respectively [54], supporting the notion that MFN proteins contribute at least indirectly to antiviral immunity.

Some publications support the presumption that peroxisomal MAVS fulfills unique functions [46,55], while this perception is questioned by others [56]. Odendall et al. observed that in Huh-7 cells, efficient type III IFN expression was only induced from peroxisomal MAVS. Additionally, they presumed a connection between peroxisome formation and type III IFN expression since peroxisome accumulation during IEC differentiation correlated with elevated type III IFN expression in response to stimulation. Conflicting with this report, Bender et al. were not able to reproduce the distinct signaling outcome of peroxisomal and mitochondrial MAVS and found that in Huh-7 cells, type I and III IFN expression is independent of the subcellular localization of MAVS.

On top of that, the perception that mitochondria as standalone organelles provide the platform for MAVS signalosome formation was thoroughly questioned recently [57]. Esser-Nobis et al. re-assessed MAVS intracellular distribution using high-resolution biochemical fractionation. They proceeded to analyze the three most relevant fractions, (1) mitochondria, (2) the microsome fraction containing ER, ribosomes, Golgi, endosomes, and lysosomes, and (3) the MAM fraction comprised of mostly ER but also peroxisomes, in detail. While MAVS was present in all fractions, activated IRF3 was absent from the mitochondria fraction and found only in the microsome and MAM fractions. Additionally, all known components of the MAVS signalosome (RLRs, MAVS, TRAF2, TBK1, IRF3) were identified in the microsome, but not the mitochondria fraction, indicating that IRF3 activation occurs at ER-derived membranes and not mitochondria. Another interesting observation in this study was the translocation of LGP2 from the microsome fraction to mitochondria upon immune stimulation. Considering the suppressor function of LGP2 on RLR signaling, mitochondria might not serve as the platform for MAVS signalosome formation but rather as the site of sequestration of signaling inhibitors [57]. Further research will show whether ER-derived membranes are the true sites of MAVS-mediated IRF3 activation and, if so, why IFN expression is still highly dependent on mitochondrial dynamics.

#### 2.2.2. STING Forms Two Distinct Higher-Order Complexes for Signal Transduction and Regulation

STING plays a pivotal role in transducing signals in response to cytoplasmic viral DNA [7] (Figure 1B). In the inactive state, STING resides at the ER [58]. Although STING is presumably capable of activating the transcription factor NFκB already from the ER, its translocation to the ER-Golgi intermediate compartment (ERGIC)/Golgi is essential for STING-mediated IRF3 activation [59]. Treatment with inhibitors of the ER to ERGIC/Golgi trafficking, Shigella effector protein IpaJ, and brefeldin A, blocked TBK1 and IRF3 activation and thus abrogated signal transduction [60,61]. Transportation of STING to the ERGIC and Golgi is mediated by the secretory pathway and depends on the COP-II complex and ARF GTPases [20].

In the absence of a stimulus, several mechanisms apply to prevent STING activation and trafficking. Stromal interaction molecule 1 (STIM1), a Ca^2+^-sensitive protein, directly interacts with STING at the ER. STIM1 localization is controlled by the ER Ca^2+^ concentration. It is distributed throughout the ER when the Ca^2+^ concentration is high but exits the complex with STING and travels to ER-plasma membrane junctions when the concentration is low [62,63]. Loss of STIM1 can result in spontaneous activation of the STING-TBK1-IRF3 axis [62]. Additionally, the polymerization interface of STING is shielded by its C-terminal tail in the inactive state, and the binding of cGAMP causes profound changes in the conformation of the STING homodimer. These changes include the release of the C-terminal tail resulting in exposure of the polymerization interface [64] and assembly of STING tetramers or higher-order oligomers [58]. STING acts as a scaffold protein enabling the recruitment of TBK1 and initiating its own phosphorylation. In a process already described for MAVS, phosphorylated STING subsequently recruits IRF3 to the complex for activation by TBK1. This process presumably takes place at the Golgi [49].

Recently, viperin was described to be a further component of the STING enhanceosome, elevating type I IFN production [65]. Upon stimulation, viperin, TBK1, and STING form complexes by the direct and strong interaction of STING and viperin mediating activation of TBK1 by polyubiquitination. Those complexes were found at lipid droplets (LDs), the sites of viperin localization, and in the cytosol. Interestingly, viperin interaction with the adaptor molecule complex supports both viperin’s enzymatic activity and degradation. Which E3 ligase mediates the polyubiquitinylation of TBK1 is not known so far [65]; however, viperin was also reported to interact with TRAF6 during TLR signaling [66], positioning it as a potential candidate.

In addition to these adaptor protein complexes required for signal transduction, activation by cGAMP causes the formation of another higher-order STING assembly at the ER, the so-called STING phase-separator [67]. It is assumed that ER resident STING forms spherical ER membraneous biocondensates with a puzzle-like structure to circumvent an overreaction of the innate immune response. While IRF3 is excluded, this gel-like condensate contains both unphosphorylated STING and TBK1, indicating that the signaling molecules are confined within compacted membranes to dampen the immune response. Importantly, the STING phase-separator is distinct from the higher-order signaling complexes: in DNA virus-infected cells, the phase-separator only forms later and downregulates STING signaling, suggesting a tight spatial and temporal regulation of the STING protein pool [67].

#### 2.2.3. MyD88 and TRIF Organize Signaling Complexes at Activated TLRs

Last but not least, SMOC formation is also observed downstream of TLR signaling pathways [27] (Figure 1C). While the previously described signaling adaptors reside at membranes and are activated either through translocation of the respective PRRs or second messengers, TLRs are bound to membranes and depend on the mobility of their signaling adaptors. Five TIR domain-containing signaling adaptor proteins, which mediate signal transduction from activated TLRs, exist [68]. Of those, only MyD88 adaptor-like protein (MAL, also called TIRAP), MyD88, and TRIF, are required to forward signal transduction in response to viral triggers. The sorting and signaling adaptors TIRAP and MyD88 are required for signal transduction from TLR2 and TLR7-9, while TLR3 relies on the signaling adaptor TRIF [68]. Little is known about the TRIF SMOCs, but MyD88 SMOCs are called myddosomes [69,70], and their architecture is better studied and defined. Note that while all TLRs can activate NFκB, those TLRs that utilize TRIF as signaling adaptor can also activate IRF3 and IRF7 and thereby type I IFN production [7].

MyD88 and TRIF are presumably distributed throughout the cytosol and recruited to the TLR signaling complexes upon immune stimulation [68]. In the case of MyD88, recruitment to the TLRs is mediated by the sorting adaptor TIRAP [27], a peripheral membrane protein. Thanks to its lipid-binding domain, able to bind several phosphoinositide species, TIRAP is enriched at the cytosolic surface of the plasma membrane but also localizes to endosomes. This dual localization is essential for surveying the plasma membrane and endosomes for activated TLR complexes [70,71]. Upon receptor activation, TIRAP was shown to form co-filaments with the TLR4 TIR domains in a process that can probably be transferred to other TLRs since the residues relevant for these interactions are conserved among different TIR domains [72]. TLR-TIRAP filament formation induces MyD88 recruitment, assembly of the myddosome, and thereby TLR signaling [70,71,72].

MyD88 comprises two domains essential for its adaptor protein function in connecting the two sections of this signaling pathway. The C-terminal TIR domain of MyD88 interacts with the TIR domains of activated TLRs and TIRAP, while the N-terminal death domain (DD) recruits members of the DD containing interleukin-1 receptor-associated kinase (IRAK) family. Consistently, the myddosome was initially defined as an ordered complex consisting of 7-8 [73] or 6 MyD88 [69], 4 IRAK4 [69,70], and 4 IRAK1 or IRAK2 monomers hierarchically assembled into a helical signaling tower. The first described model for myddosome formation assumed successive incorporation of the individual components, beginning with MyD88 oligomerization at activated TLRs [69]. However, Moncrieffe et al. recently postulated a different mechanism of myddosome assembly in which the MyD88 scaffold does not form de novo at activated receptors but is pre-assembled in the cytosol, and receptor activation is only required for IRAK recruitment [74]. Importantly, once formed, myddosomes are highly stable molecular assemblies that do not exchange with un-complexed MyD88, IRAK4, or IRAK1, as demonstrated by photobleaching experiments [75]. This trait distinguishes them from liquid-like phase-separated condensates indicating that the myddosome can rather be described as a solid-like SMOC. Auto-phosphorylation of IRAK4 and subsequent phosphorylation of IRAK1/2 is most likely facilitated by the close proximity of the kinase domains within the myddosome [69,76]; nevertheless, additional ubiquitination of IRAK1 by TRAF6 is required for NFκB activation [77,78]. To mediate K63-linked ubiquitination, IRAK1 initiates the formation of a complex with TRAF6 and the LD-anchored protein viperin [79]. Although very interesting, the association with LDs raises more questions. So far, it is not quite understood what happens to the myddosome after assembly. IRAK1 and IRAK2 might leave the complex post-phosphorylation [69] to interact with the downstream signaling components viperin and TRAF6. However, this perception is in conflict with the occasional co-localization of viperin-positive LDs and MyD88 [66], which could suggest at least sporadic recruitment of whole myddosomes to LDs. In addition, viperin also co-localizes with IRF7 and facilitates its nuclear translocation in response to TLR9 elicitation by CpG DNA. Although not well established, viperin seems to be a crucial component of MyD88 mediated signaling, as demonstrated by impaired IFN-β production in response to TLR7 and TLR9 activation in viperin knockout DCs [66]. This observation and the interaction of viperin with the STING signaling complex (see above) might lay the foundation for further research investigating the contribution of the so far under-appreciated LDs to innate immune signaling.

In contrast to the myddosome, the signalosome surrounding TRIF is not well studied. Nevertheless, higher-order assemblies nucleated by TRIF are known to exist. Upon TLR3 activation, TRIF transiently co-localizes with the receptor and then proceeds to form speckle-like signalosomes in the cytoplasm that contain TBK1 [80]. In addition to NFκB, which is activated by all named TLRs, TLR3 signaling also activates the transcription factor IRF3 in a mechanism similar to MAVS and STING [49].

## 3. Viral Antagonism of IFN Signaling Via Re-Localization of Host Factors

**Figure 2:**
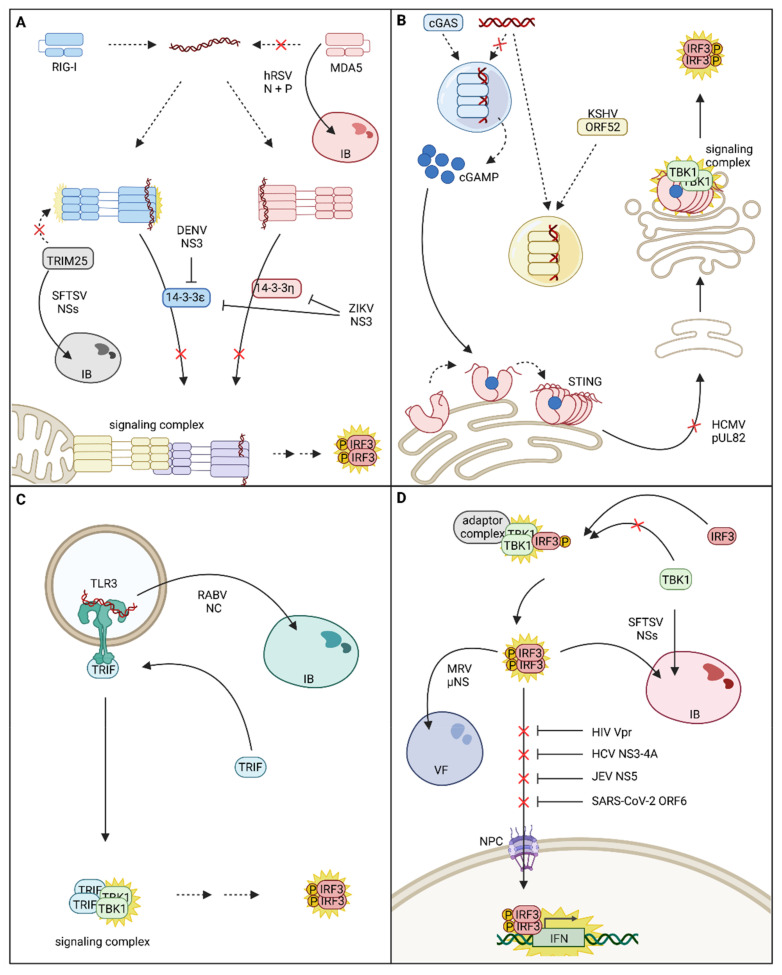
Viral strategies for disruption of innate immune signaling compartmentalization and spatial dynamics. (**A**) Spatial interference with the melanoma differentiation gene 5 (MDA5)/retinoic acid-inducible gene-I (RIG-I)-mitochondrial antiviral signaling (MAVS) signaling cascade: human respiratory syncytial virus (hRSV) N and P proteins form inclusion bodies (IBs) within which MDA5 is sequestered, thus impairing MDA5 mediated activation of innate immune signaling. The severe fever with thrombocytopenia syndrome virus (SFTSV) NSs protein evolved to sequester tripartite motif containing 25 (TRIM25) into virus-induced IBs and prevents full activation of RIG-I and RIG-I mediated signal transduction. Zika virus (ZIKV) and Dengue virus (DENV) NS3 evolved to inhibit the chaperone-mediated translocation of activated MDA5 and/or RIG-I to their signaling adaptor MAVS and interrupt signal transduction. (**B**) Spatial interference with the cyclic GMP-AMP synthase (cGAS)-stimulator of interferon genes (STING) signaling cascade: The Kaposi’s sarcoma-associated herpesvirus (KSHV) tegument protein ORF52 forms its own liquid-like organelles with viral DNA and is even capable of extracting viral DNA from phase separations with cGAS, inhibiting the activation of cGAS and production of the second messenger cGAMP. Another strategy for inhibiting cGAS-STING signaling is the prevention of STING trafficking, as demonstrated by human cytomegalovirus (HCMV) pUL82. (**C**) Re-localization of Toll-like receptor 3 (TLR3): rabies virus (RABV) NC protein was shown to incorporate TLR3 into viral IBs; however, the effect of TLR3 sequestration on RABV-induced interferon (IFN) expression was not investigated so far. (**D**) Inhibition of IFN regulatory factor 3 (IRF3) nuclear translocation: Translocation of activated IRF3 into the nucleus is impeded by several viruses. SFTSV sequesters both TANK-binding kinase 1 (TBK1) and activated IRF3 into IBs to prevent IFN expression. Likewise, mammalian reovirus (MRV) µNS is able to sequester activated IRF3 into viral factories (VFs). Human immunodeficiency virus (HIV), hepatitis C virus (HCV), Japanese encephalitis virus (JEV), and SARS-Corona virus-2 (SARS-CoV-2) interrupt nuclear translocation of IRF3 by interference with the nuclear import machinery (nuclear pore complex (NPC)). Within this figure, re-localization events are indicated with continuous arrows, and activation events are indicated by yellow stars. Red crosses indicate re-localization events that are impaired by viral immune evasion strategies.

A prerequisite for viral replication is the inhibition of a strong antiviral immune response by evasion from immune recognition or disruption of signal transduction. Successfully replicating viruses relyon a plethora of strategies to dampen the antiviral immune response, including shielding of viral genomes to prevent PRR sensing, cleavage or degradation of signaling molecules, and inhibition of posttranslational modifications required for signal transduction [9,81]. In this review, we want to focus on another subset of viral immune evasion strategies, interfering with the intracellular localization of signaling effectors, either by inhibition of correct localization or sequestration to compartments that do not support signal transduction (Figure 2).

### 3.1. PRR Re-Localization Facilitates Viral Immune Evasion

Replication of many viruses induces the formation of specialized subcellular compartments, called viral factories. These viral factories often concentrate viral replication factors, provide platforms for replication and assembly, and shield PAMPs from immune recognition. They can either be associated with membranes, as is the case for many positive-sense ssRNA viruses or be membrane-less [8]. Membrane-less viral factories of negative-sense ssRNA viruses are called inclusion bodies (IBs) and often exhibit liquid-like properties [82]. Besides their important role in facilitating propagation, IBs also equip the virus with a segregated subcellular compartment suitable for the sequestration of innate immune signaling proteins. In human respiratory syncytial virus (hRSV) infected cells, MDA5 is sequestered within viral IBs, and the presence of IBs alone, induced by overexpression of hRSV N and P proteins, is sufficient to target MDA5 into viral IBs (Figure 2A). The sequestration of MDA5 is most likely ruled by the direct interaction of hRSV N and MDA5 and results in decreased IFN-β expression in response to a viral stimulus [83]. Another virus that sequesters PRRs into IBs is severe fever with thrombocytopenia syndrome virus (SFTSV), a member of the Bunyavirales (Figure 2A). Co-immunoprecipitation experiments revealed that SFTSV NSs interacts with TBK1 [84], TRIM25 [85,86], and RIG-I, although RIG-I co-precipitation was probably mediated by indirect interactions through TRIM25 [87]. NSs’ interactions result in the sequestration of TBK1, TRIM25, and RIG-I into viral IBs and thus inhibit activation of the IFNB promoter in response to viral infection or treatment with dsRNA [87]. Min et al. further highlighted the relevance of the RIG-I signaling pathway for the SFTSV-mediated immune responses, and although they could not reproduce NSs interaction with RIG-I, they confirmed its direct interaction with TRIM25 and its recruitment into SFTSV-induced IBs. Further studies will be necessary to determine whether or not RIG-I is sequestered into SFTSV IBs. Nevertheless, sequestration of TRIM25, required for RIG-I posttranslational modification, might already be sufficient to interrupt the RIG-I signaling pathway and prevent adverse effects of innate immunity on viral replication.

Other strategies to impair RNA recognition by cytoplasmic PRRs are disruption of stress granules, which contain MDA5 and RIG-I, by encephalomyocarditis virus and poliovirus [86,88] or inhibition of trafficking. Thus, Dengue virus (DENV) NS3 was shown to directly bind 14-3-3ε and impair its interaction with RIG-I. This results in the suppression of RIG-I translocation to sites of MAVS localization and thereby blocks signal transduction and alleviates IFN-β and cytokine expression [9]. Mechanistically, DENV NS3 harbors a RxEP motif that mimics the phosphorylable motif (Rxx(pS/pT)xP) used by host factors to bind 14-3-3 proteins. Notably, in the viral protein, the charged glutamic acid residue (E) replaces the canonical phosphorylable threonine or serine residue. This phosphomimetic residue is likely an advantage to outcompete the RIG-I binding to 14-3-3ε, which is sensitive to dephosphorylation. Strikingly, this phosphomimetic motif is conserved in West Nile and Zika viruses (ZIKV) (RLDP motif) and was shown, in the case of ZIKV, to inhibit not only RIG-I signaling via binding of 14-3-3ε but also MDA5 signaling via binding of the corresponding 14-3-3η chaperone [89] (Figure 2A).

For DNA viruses, recognition of the viral genomes by cGAS and activation of the associated signaling cascade pose a major threat. Therefore, herpesviruses express a highly conserved tegument protein [90,91] which counteracts cGAS activity and enables evasion from immune recognition [92]. In fact, just like cGAS, the γ-Herpesvirus Kaposi’s sarcoma-associated herpesvirus (KSHV) protein ORF52 forms liquid-like condensates with DNA [90,91] in a length-dependent but sequence-independent manner [90]. Astonishingly, ORF52 seems to have a stronger affinity to cytoplasmic DNA than cGAS and is, therefore, able to outcompete the PRR [91]. ORF52 was found to accumulate around cGAS-DNA condensates and gradually extract DNA from liquid droplets. ORF52 then continues to form its own condensates with the extracted DNA, resulting in the collapse of cGAS-DNA phase separation [91] (Figure 2B). In agreement with the fact that DNA sensing by cGAS needs to be inhibited immediately upon release of the viral genome, inhibition of cGAS activation occurs very early during viral infection and is mediated by ORF52 proteins introduced with the invading virion [91]. Besides ORF52 from KSHV, homologs from other γ-herpesviruses [90,91] and also α-herpesviruses fulfill the same function [91].

Additionally, some viruses have evolved not only to re-localize PRRs but also to simultaneously exploit them by utilization of non-canonical, pro-viral functions. Rabies virus (RABV) infection induces the formation of cytoplasmic aggregates, resembling IBs, which are called Negri bodies (Figure 2C). Interestingly, TLR3 was found to be concentrated within those Negri Bodies together with the viral nucleocapsid protein NC and viral genomic RNA. Although TLR3 and NC do not directly interact, TLR3 is crucial for Negri body formation since Negri bodies do not form in TLR3 deficient cells. Furthermore, the absence of TRIF in Negri bodies leads to the assumption that TLR3 is not activated or signaling competent within the virus-induced compartment [93].

### 3.2. Viral Interference with Adaptor Protein Localization

While sensing of viral genomes can be mediated by a variety of different proteins, not all of which are mentioned in this review, the signaling cascades converge at the level of signaling adaptors. Therefore, interference with adaptor localization or manipulation of signaling relevant organelle integrity is a strategy often applied by viruses to impair signal transduction.

#### 3.2.1. Interference with STING Subcellular Localization

STING is the adaptor of the cGAS-associated signaling pathway and thus links cGAS DNA sensing to the activation of the transcription factor IRF3 via phosphorylation by TBK1. As already explained previously, the trafficking of STING from ER to the ERGIC/Golgi compartment is essential for successful signal transduction. Therefore, this process poses a potential target for viral immune evasion strategies. The HCMV tegument protein pUL82 was shown to interfere with STING intracellular trafficking, resulting in inhibition of STING signaling complex assembly. This impairs the phosphorylation of TBK1 and IRF3 and consequently dampens IFN-β expression [94] (Figure 2B). HCMV further exploits STING to facilitate nuclear entry. Early post infection, STING binds to the viral capsid protein and mediates co-localization of the viral capsid with nuclear pore complexes, resulting in nuclear accumulation of viral genomes. In turn, STING deficiency inhibits the import of viral genomes into the nucleus. Additionally, in monocytes, an HCMV reservoir, STING promotes the establishment of HCMV latency and reactivation. Mediation of nuclear import requires correct localization of STING at the ER [95,96], and one could assume that the previously mentioned mechanism for inhibition of STING trafficking might directly or indirectly contribute to the nuclear import of HCMV genomes. STING localization is also thoroughly manipulated by rhinoviruses (RVs). Interestingly, STING was identified as an RV-A2 host protein in a genome-wide siRNA screen; however, canonical STING activation via cGAMP was not required for its RV-A16 host factor activity [96]. RVs are RNA viruses, and as expected, the immune response against RVs is primarily mediated via RIG-I and MDA5 signaling pathways and does not rely on STING [97]. Triantafilou et al. found that in air–liquid interface cultures of primary human airway epithelial cells, canonical STING function was impaired during RV infection, but STING expression was significantly upregulated. In agreement with the inability of STING to fulfill its canonical function during RV infection, STING was also not following its canonical trafficking route but localized to viral replication organelles [96,97]. This non-canonical localization is partially mediated by the RV 2B protein, which reduces the ER Ca^2+^ levels and induces the release of STING from STIM1 [97]. In addition to its role as an adaptor of the DNA sensing pathway, STING crosstalks with RLR signaling and potentiates RIG-I-mediated IFN induction [98]. Although in the published setting, STING knockout did not affect IFN production after RV infection [97], in other cell systems, co-opting STING as a host factor could further alleviate the cell defenses to infection.

#### 3.2.2. Interference with Mitochondrial Dynamics Impairs IFN-β Expression

Mitochondria are highly dynamic organelles and are still widely accepted as the site of MAVS localization. As explained previously, mitochondrial fission and fusion events have a direct impact on innate immune signaling via MAVS. However, dynamic fission and fusion between mitochondria and constant mixing of mitochondrial content require preservation of mitochondrial fitness by extraction of partially defective mitochondria from the pool. Mitochondrial fitness is maintained by mitophagy, which constitutes a specific form of autophagy. Mitophagy is either receptor- or ubiquitin-mediated, for example, by the E3 ubiquitin ligase Parkin, induces the formation of autophagosomes around mitochondria, and culminates in delivery to and fusion with lysosomes for degradation. In addition to the regulation of inflammatory responses, mitophagy also plays a role in other essential cellular processes [99]. A multitude of viruses was shown to deliberately induce mitophagy to inhibit apoptosis and thereby promote viral replication [100,101,102]. However, in the following chapter, we want to specifically focus on those events of virus-induced mitophagy that were also shown to contribute to immune evasion.

Measles virus (MeV) induces mitophagy, as attested by the engulfment of mitochondria in autophagosomes and the drastic reduction in mitochondrial mass in infected cells. SiRNA targeting of essential autophagy effectors in this infection setting resulted in higher expression levels of IFN-β and, therefore, stronger elicitation of the innate immune response. Additionally, autophagy effector KO cells had higher MAVS expression in response to MeV infection than wildtype [103], emphasizing the relevance of mitochondrial degradation for MeV immune evasion. The SARS-CoV-2 ORF10 protein also interferes with the mitochondrial dynamics by induction of mitophagy. ORF10 overexpression reduces MAVS expression in a dose-dependent manner and attenuates type I IFN expression in response to the elicitation of MAVS-mediated signaling. These immune inhibitory effects can be counteracted by treatment with autophagy inhibitors, directly linking ORF10-mediated immune evasion to mitophagy [104]. Coxsackievirus B3 (CVB3) induces Parkin-dependent mitophagy in a range of host cells to promote viral replication. This can be blocked by inhibition of Drp1 mediated mitochondrial fission, a process required for mitophagy. Consistent with that, CVB3 infection in Drp1-deficient cells resulted in stronger activation of TBK1 and higher IFN-β expression, and chemical induction of mitophagy supported viral replication and reduced IFN expression [105]. Influenza A (IAV) alternate reading frame protein PB1-F2 localizes to the mitochondrial inner membrane space in close association with the inner membrane, where it forms assemblies of three or more monomers. PB1-F2 attenuates the mitochondrial inner membrane potential, a process capable of triggering mitophagy [99], and causes enhanced mitochondria fragmentation. PB1-F2 expression was sufficient to inhibit MAVS-dependent activation of the RIG-I signaling pathway in a dose-dependent manner and to efficiently inhibit the phosphorylation of IRF3 in response to poly(I:C) elicitation [106]. More recently, it was shown that the activity of PB1-F2 relies on its interaction with the mitochondrial protein TUFM, which is required for PB1-F2 mediated mitophagy and subsequent attenuation of the antiviral immune response [107]. Interestingly, some viruses seem to promote incomplete mitophagy by strict regulation of mitophagy progression. Human parainfluenza virus type 3 (HPIV3) induces incomplete mitophagy with the help of two viral proteins exhibiting distinct functions. Expression of the viral M protein causes complete mitophagy via interaction with TUFM and LC3, and overexpression of M results in a downregulation of IFN expression raised in response to stimulation with Sendai virus (SeV). However, HPIV3 P suppresses the fusion of mitophagosomes and lysosomes and thus hinders complete mitophagy. According to the authors, this inhibition of complete mitophagy might be required to provide membranous assembly and transportation platforms for virus replication [108,109]. Whether or not complete mitophagy is induced by HCV infection is still a matter of debate [110]. On the one hand, HCV is capable of inducing Drp1-dependent induction of mitochondrial fission, and higher levels of activated Drp1 were found in livers of chronically HCV-infected patients, highlighting the biological relevance of this process. Besides the assumption that induction of mitophagy inhibits apoptosis and consequently favors HCV persistence, interference with HCV-mediated mitochondrial fission also enhances expression from an IFN-stimulated promoter, suggesting enhanced IFN production [111]. The described HCV-induced changes in mitochondrial dynamics can likely be attributed to HCV NS5A since its overexpression alone is sufficient to cause complete autophagy [110]. On the other hand, HCV core was found to exhibit an opposite effect on mitophagy. HCV core interacts with Parkin and suppresses its translocation to mitochondria, resulting in inhibition of mitophagy [110,112]. This indicates that HCV itself might strictly regulate the progression of mitophagy. The hypothesis that HCV induces incomplete mitophagy is further supported by the observation that HCV indeed induces the accumulation of autophagosomes but does not culminate in enhanced protein degradation [113]. Wang et al. demonstrated that HCV induces the expression of two distinct host proteins with opposite functions in autophagy, one inhibiting and one stimulating the maturation of autophagosomes. These two proteins are consecutively upregulated, and expression of the stimulator of autophagosome maturation is delayed, resulting in the accumulation of autophagosomes in early infection stages and later on induction of degradation [114]. How incomplete mitophagy or differential control of mitophagy progression contributes to viral replication needs to be further investigated. Nevertheless, mitophagy could support viral replication with functions distinct from inhibition of apoptosis or facilitation of immune evasion.

While all previous examples focused on virus-induced fragmentation of mitochondria, eventually resulting in mitophagy to suppress MAVS activation, DENV and ZIKV pose a very interesting exception to that common theme. DENV virus infection induces major membrane alterations and the formation of an ER-derived membraneous compartment consisting of several substructures, one of which is convoluted membranes (CMs) [115]. Instead of activating Drp1 to induce mitochondrial fission, DENV inhibits Drp1 activation, and DENV and ZIKV infection, as well as expression of DENV NS4B alone, induce mitochondrial elongation. However, while in uninfected cells, mitochondria are in contact with MAMs, this interaction is disrupted by DENV infection. DENV-induced CMs connect to mitochondria and thereby cause the loss of the mitochondria–ER interface. Mitochondria elongation promotes DENV and ZIKV replication and attenuates DENV-induced innate immunity by a decreased RIG-I abundance in the MAM fraction [116]. This immune evasion strategy impressively demonstrates that not only the spatial organization but also organelle interaction is crucial for successful immune signaling.

### 3.3. Viral Interference with Transcription Factor Nuclear Translocation

One of the very last opportunities for a virus to circumvent immune activation is interfering with the translocation of transcription factors into the nucleus (Figure 2D). Thus, a number of viruses have evolved strategies to prevent the translocation of activated transcription factors by sequestration of transcription factors in cytoplasmic compartments or interfering with the nuclear import machinery. Mammalian reovirus (MRV) impedes the nuclear translocation of already active IRF3 by retaining the transcription factor in cytoplasmic viral factories. Sequestration of IRF3 is solely mediated by the MRV µNS protein, and the expression of µNS alone is sufficient to suppress the translocation of IRF3 in response to immune stimulation and alleviate the IFN response [117]. As already indicated earlier, SFTSV is an expert in the sequestration of immune factors, and these processes mainly rely on the activity of the NSs protein. NSs was shown to interact with TBK1, which facilitates the incorporation of TBK1, IRF3, and the IKK complex into viral IBs [118,119]. Since activated IRF3 is captured within IBs, it is hindered from activating IFN expression due to impaired nuclear translocation. Consequently, NSs expression and sequestration of IRF3 reduce the IFN response [118]. Furthermore, also activated IRF7 was shown to be imprisoned in viral IBs, in this case probably due to direct interaction with NSs. Sequestration of IRF7 is beneficial for the virus because it reduces the induction of the type I IFN response and enhances viral replication [120].

Targeting the nucleocytoplasmic transport machinery is a further option to prevent the nuclear translocation of the IRF3 and IRF7 transcription factors. Macromolecule exchange across the nuclear envelope takes place at the nuclear pore complex and is mediated by nuclear transport receptors, also called importins or karyopherins (KPNAs). This process is manipulated by DNA viruses and some RNA viruses to access their nuclear replication compartment, but also by viruses of distinct families to counteract innate immune signaling [121]. Among the Flaviviridae, the HCV protease NS3-4A is able to block IFN production at several levels. Best known to cleave MAVS, NS3-4A also interacts with the nucleocytoplasmic transport [122] and cleaves importin-β1 (KPNB1), impeding the nuclear translocation of IRF3 and NFκB [123]. NS5 protein from the Japanese Encephalitis virus also blocks poly(I:C)-triggered IFN production. NS5 interacts with importin-α3 and α4 (KPNA4 and 3, respectively) via its nuclear localization signal and competes in a dose-dependent manner with the binding of activated IRF3 and NFκB, preventing their nuclear translocation. Overexpression of importin-α3 or α4 relieves the blockade and rescues IFN production [124]. A similar competition mechanism was more recently described for HIV-1 Vpr. The accessory protein Vpr is essential for HIV-1 replication in human monocyte-derived macrophages when the DNA sensing pathway is activated, e.g., upon cGAMP treatment. Kahn and colleagues demonstrated that Vpr inhibits innate immune activation and IFN production triggered by a panel of stimuli by preventing IRF3 nuclear translocation downstream of its activation. Vpr localizes to the nuclear pores, binds importin-α5 (KPNA1), and prevents the recruitment and translocation of IRF3 and NFκB [125]. Finally, among the panoply of strategies displayed by SARS-CoV-2 to suppress IFN induction, a similar mechanism was suggested for ORF6, which binds importin-α1 (KPNA2) and prevents IRF3 nuclear translocation [126].

## 4. Conclusions

In conclusion, the cellular innate immune response upon encounter with pathogens is highly organized in time and space. Signal transduction from the pathogen detection sites to the promoters of antiviral genes involves intracellular membranes to concentrate the adaptor platforms and spans multiple organelles and membrane-less compartments. Several of the involved players also shuttle between signaling and repository sites. In addition to innate immune players, which might change location during the elicitation of the defense mechanisms, whole organelles can be remodeled, as seen, for instance, with the mitochondrial elongation observed upon RLR activation [51]. The cell cytoskeleton is often manipulated upon viral infections and contributes to the formation of the viral replication organelles [8]; its role in innate immune compartmentalization is also an interesting topic that we did not address in this review. Strikingly, with up to 10% of the human genes prone to be regulated by IFNs [6], innate immune elicitation initiates a dramatic event for the host cell. Proteomics-based methods will be useful to assess the compartmentalization and the spatial reorganization of the cell proteome during the IFN production and response cascades in an unbiased manner and on a system-wide scale. Thus, while major protein translocations in the cascades rely on known phosphorylation events (e.g., governing IRF3 nuclear translocation), phosphoproteomics might uncover new concurrent dynamic events [127]. Furthermore, protein correlation profiling [128] has opened the possibility of interrogating the proteome of all major cellular organelles in parallel and could provide a detailed map of the cell responding to a viral insult. Finally, the spatial organization of the innate antiviral immune response at the level of the cell population adds another layer of complexity to the intracellular compartmentalization described in this review. Spreading of signaling molecules to bystander cells (e.g., transfer of cGAMP via gap junctions [129]) and unequal IFN production and response (as reviewed in [130]), underlined by a combination of complex gene expression patterns and stochastic responses, also spatially regulate the antiviral response among seemingly homogenous cell populations, possibly preserving the balance between efficient pathogen defense and proper maintenance of cell physiology.
